# Incidence and attributes of chimeric COI and 18S rDNA sequences derived from nematode-infected arthropods

**DOI:** 10.1186/s12864-026-13235-3

**Published:** 2026-08-05

**Authors:** Jacopo D’Ercole, Robin Floyd, Sean WJ Prosser, Saeideh Jafarpour, Paul DN Hebert

**Affiliations:** 1 Centre for Biodiversity Genomics, University of Guelph, Guelph, Ontario Canada; 2 Department of Integrative Biology, University of Guelph, Guelph, Ontario Canada; 3 Department of Chemistry and Biology, Toronto Metropolitan University, Toronto, Ontario Canada

**Keywords:** PCR, Chimeric sequences, DNA barcoding, COI, 18S rDNA, HTS, High-throughput sequencing, Oxford Nanopore

## Abstract

**Background:**

PCR amplification of mixed-template samples can generate chimeric sequences, molecules that combine partial sequences from two or more source organisms. These artefacts pose a significant challenge for high-throughput sequencing studies, generating both type I errors (true species mistakenly rejected) and type II errors (chimeras incorrectly viewed as valid species). Despite their impact, the incidence, structural properties, and factors driving chimera formation remain poorly understood.

**Results:**

Using nanopore sequencing of 531 nematode-infected arthropod specimens, chimera formation was characterized in two widely used gene regions: the 658 bp of cytochrome *c* oxidase subunit I (COI) barcode and a 900 bp segment of 18S ribosomal DNA (18S rDNA). Chimeras were detected for both gene regions despite the deep sequence divergences between members of these two phyla. However, the incidence of chimeric molecules was higher for 18S rDNA than COI with chimera formation correlating with local variation in DNA stability and conserved sequence tracts between parent species.

**Conclusions:**

By clarifying the associations between DNA stability, sequence conservation, and chimera formation, this study offers insights that can inform the development of more robust approaches to chimera detection.

**Supplementary Information:**

The online version contains supplementary material available at https://doi.org/10.1186/s12864-026-13235-3.

## Background

DNA barcoding has been widely adopted for specimen identification [1] and species discovery [2, 3]. These same features also make it a powerful tool for describing [4–7] and monitoring [8–10] genetic diversity. High-throughput sequencing (HTS) has advanced the application of DNA-based identification systems by making it possible to ascertain the species composition of bulk and environmental samples (i.e., metabarcoding and eDNA) [11–13], and to reveal species interactions (i.e., symbiome) [14, 15]. However, the detection of rare species in both contexts is complicated because the misinterpretation of sequence data can create both type I errors (where valid species are mistakenly rejected) and type II errors (where sequencing artifacts are viewed as true species).

Chimeric amplicons are prime candidates for misinterpretation because they combine partial sequences from two or more species. Despite this fact, their incidence, structural properties, and impact on DNA barcoding/metabarcoding have seen limited investigation. Most chimeras arise when the elongation of an amplicon terminates prematurely, creating a truncated product which can itself serve as a primer in subsequent PCR cycles. When an incomplete product from one species binds to template from a second taxon and extension proceeds, it produces an interspecific chimeric sequence [16–18]. Their presence creates two primary issues. First, chimeric sequences inhibit the amplification of target template molecules. Functioning as DNA templates themselves, they compete for reagents during amplification, reducing PCR efficiency [19]. Second, chimeric sequences are distinct from both of their parent taxa, and, if not recognized, can be misinterpreted as representing new species [20, 21]. Therefore, it is important to characterize chimeric molecules and identify the factors that influence their formation.

This study examines sequence variation in two gene segments commonly used to assess species diversity, the barcode region of the cytochrome *c* oxidase subunit I (COI) and a segment of the 18S ribosomal DNA (18S rDNA). It examines the incidence and structure of chimeric molecules created when DNA extracts derived from nematode-infected arthropods are PCR amplified. Although no primers are universal, the COI [22] and 18S rDNA [23] primer pairs used here target highly conserved regions of these genes that have been shown to amplify across a wide range of metazoan taxa, including both arthropods and nematodes [24, 25], meeting the basic requirement to generate chimeric sequences.

## Methods

### Study design

As part of the international DNA barcoding initiative led by the Centre for Biodiversity Genomics, the Sequel and Sequel II platforms (Pacific Biosciences) have been used to sequence COI amplicons from approximately 10 million DNA extracts, each derived from a single arthropod specimen. These extracts were generated across numerous independent projects over the past decade. Inspection of the sequence arrays from a subset of 0.1 million of these specimens revealed 531 cases where both COI arthropod and nematode sequences were recovered from the same individual, indicating that the arthropod carried a nematode infection. These extracts, derived from nematode-infected arthropods, provided a perfect opportunity to assess the incidence and impact of chimeric molecules on these two genetic markers. To this end, COI was reamplified and 18S rDNA was amplified de novo, and both were sequenced.

A pairwise comparison of the arthropod and nematode sequences from these 531 specimens revealed an average divergence of 34% for COI and 24% for 18S rDNA. Because of this high divergence, chimeras between arthropod and nematode sequences are easily recognizable.

### Sampling and genetic characterization

The 531 arthropods included 527 insects and 4 arachnids. The 658 bp barcode region of mitochondrial COI and approximately 900 bp of 18S rDNA were amplified with Platinum Taq (Thermo Scientific) using standard primers: C_LepFolF/C_LepFolR [22] for COI and SSU_F_07/SSU_R_26 [23] for 18S rDNA. Primers were asymmetrically indexed with unique molecular identifiers (UMIs) to allow the pooling of PCR products from all 531 specimens in a single sequencing run. Primer and UMI sequences are provided in Supplementary Table 1. Each PCR included: 6.25 µl of 10% trehalose (Fluka Analytical), 1.62 µl of molecular grade water (Hyclone), 1.25 µl of 10X Platinum Taq buffer (Thermo Scientific), 0.625 µl of 50 mM MgCl_2_ (Thermo Scientific), 0.0625 µl of 10 mM dNTPs (KAPA Biosystems), 0.625 µl of 2 µM forward primer (IDT), 0.0125 µl of 100 µM reverse primer (IDT), 0.06 µl of 5 U/µl Platinum Taq (Thermo Scientific), and 2 µl of DNA for a total reaction volume of 12.5 µl. PCR for COI employed 45 cycles with the following regime: 94 °C for 2 min; 5 cycles of 94 °C for 40 s, 45 °C for 40 s, 72 °C for 1 min; 40 cycles of 94 °C for 40 s, 51 °C for 40 s, 72 °C for 1 min; 72 °C for 2 min. PCR for 18S rDNA employed 35 cycles with the following regime: 94 °C for 2 min; 35 cycles of 94 °C for 40 s, 56 °C for 40 s, 72 °C for 1 min; 72 °C for 5 min.

Library preparation for both genes followed ONT recommendations. Briefly, each amplicon pool underwent end preparation using the NEBNext Ultra™ II End Repair/dA-Tailing Module (New England Biolabs, NEB), followed by purification with AMPure-XP beads. Next, ONT sequencing adapters (SQK-LSK114) were ligated onto the amplicons using NEB Quick T4 DNA Ligase (NEB), followed by a final purification step with AMPure-XP beads. Sequencing was performed on a Mk1b ONT sequencer using either a MinION (FLO-MIN114, version 10.4.1) or a Flongle (FLO-FLG114, version 10.4.1) flow cell. The COI library was analyzed on a single MinION flow cell, while the 18S rDNA library was analyzed on two Flongles which were combined before further analysis. The higher read depth provided by the MinION flow cell was essential for COI, as preliminary analysis of the Sequel data only detected 27 COI chimeric sequences across the 531 samples (data not shown), highlighting the need for increased sequencing depth. Although the MinION and Flongle flow cells differ in pore number and therefore in throughput, both employed identical R10.4.1 chemistry and were therefore expected to yield comparable per-read error profiles.

### Bioinformatic pipeline for high-throughput sequencing

Sequence analysis was performed in an Ubuntu environment (22.04.3 LTS) employing the informatics pipeline outlined by Hebert et al. [26]. This pipeline involved base calling, demultiplexing, filtering reads by the length of the target amplicon, clustering reads into OTUs, and assigning taxonomy to the OTUs. The MinION flow cell generated 2,345,412 reads, of which 1,486,303 were retained after filtering. By comparison, the two Flongles produced 1,071,419 reads, with 187,183 retained after filtering. The full COI dataset derived from the MinION flow cell was employed for comprehensive examination of the chimeras derived from COI amplicons. However, to enable a direct comparison of the incidence of chimeras between COI and 18S rDNA, the MinION dataset was haphazardly downsized to match the read count achieved by the two Flongles (i.e., 187,183 reads).

### Chimera detection

Three complementary approaches were applied to assess the robustness of chimera detection. For direct comparison between the two markers, all three approaches were applied to the reduced COI dataset and the 18S rDNA dataset, both equal in sample size.

The first approach assessed robustness of chimera detection through systematic one-at-a-time variation of parameters in the *uchime_denovo* algorithm. The *uchime_denovo* command in VSEARCH v2.30.2 [27] identifies chimeric sequences by dividing each query into four non-overlapping segments, which are used to search for candidate parent sequences among more abundant OTUs [28]. A three-way alignment is constructed between the query and the two best candidate parents, and a chimera score is computed from nucleotide differences. The algorithm scores each query sequence against candidate parents using four parameters: (i) *minh*, the minimum chimera score (H score) required to flag a sequence as chimeric, reflecting overall confidence in the chimeric assignment; (ii) *abskew*, the minimum abundance ratio required between each parent sequence and the chimera, based on the expectation that chimeric artefacts are less abundant than their template sequences; (iii) *mindiffs*, the minimum number of nucleotide differences required between each segment of the chimeric sequence and the parent it does not derive from; this threshold ensures that the two arms are distinct enough from each other’s alternative parent to be confidently attributed to separate parental sequences; and (iv) *mindiv*, the minimum overall sequence divergence required between the chimera and its best-matching single parent, preventing highly similar sequences from being classified as chimeras. An initial analysis ran the four detection parameters at their default values (*minh* = 0.28, *abskew* = 2.0, *mindiffs* = 3, *mindiv* = 0.8). Each parameter was subsequently tested independently at two alternative values (*minh*: 0.1 and 0.5; *abskew*: 1.2 and 4.0; *mindiffs*: 1 and 5; *mindiv*: 0.3 and 1.5) while holding all other parameters at their default values. This one-at-a-time design yielded nine configurations in total and allows the contribution of each parameter to chimera detection to be assessed independently.

The second approach consisted of manual chimera screening performed independently of algorithmic predictions through visual inspection of aligned sequences. This process consisted of four steps. First, for each sample containing three or more OTU sequences (the minimum required to identify a chimera and its two parent sequences), all sequences were aligned using MAFFT v7.490 [29]. Second, aligned sequences were inspected for transitions in nucleotide identity patterns characteristic of chimeric molecules. Third, when more than two potential parent sequences were identified for a given chimera, only the two most abundant sequences (based on read counts) were retained as candidate parents, reflecting the higher probability that abundant templates serve as parents in chimera formation. Fourth, crossover point identification was restricted to chimeras exhibiting a single recombination event, as these represent the predominant form in amplicon datasets and, unlike multiple-crossover chimeras, can be unambiguously detected through visual inspection [18].

The third approach used the experimental *chimeras_denovo* command in VSEARCH v2.30.2 to address two related questions: whether the number of detected chimeras changes with increasing segmentation, and whether perceived crossover locations shift as segment boundaries change, particularly with respect to the observed absence of crossovers near sequence termini. For COI sequences (~ 650 bp), 4, 6, and 8 segments were tested (approximately 163, 108, and 81 bp per segment, respectively). For 18S rDNA sequences (~ 900 bp), identical segment configurations were tested (approximately 225, 150, and 113 bp per segment, respectively). All other parameters were held constant (*chimeras_diff_pct* = 0.0, *chimeras_length_min* = 50, *chimeras_parents_max* = 3).

For the reduced COI dataset, the 18S rDNA dataset, and the full COI dataset, the final group of high-confidence chimeras was defined as the intersection of *uchime_denovo* detections at default settings and independent visual inspection. The default parameter configuration was selected as it offered a balance between sensitivity and specificity that minimized both false negatives and false positives, a choice consistent with a recent benchmark showing that *uchime_denovo* at default settings achieved higher precision than newer, long-read-specific alternatives, including *chimeras_denovo* [30]. This conservative approach favors a reliable dataset of unambiguous chimeras, providing a solid basis for determining crossover locations and conducting all derived analyses.

### Statistical analysis

Histograms displaying sequence recovery for the two gene regions, along with those illustrating the distribution of sequences per sample, were generated using the *ggplot*2 ver. 3.5.3 [31] package in R Studio.

Hartigan’s Dip Test [32] was performed with the *diptest* ver. 0.77.1 package on R Studio to assess the unimodality of the distribution of midpoint values of crossover ranges. This was followed by the application of a Kernel density estimate, using the package *KernSmooth* ver. 2.23.26 to create a smooth representation of the density. This integrated approach of statistical testing and visualization provided a robust method for evaluating the unimodal characteristics of the dataset.

A custom R script (Supplementary Script 1) was used to visualize the crossover range of the chimeric molecules characterized by a single crossover region. Chimeric sequences were grouped according to the taxonomic status of their parent sequences. They were classified into three categories (Unknown, Same, Mixf) based on whether they originated from an unknown phylum, from the same phylum, or different phyla. The graphical representation was generated using the package *ggplot2* [31] in R Studio.

Analysis of variance (ANOVA) was conducted using R Studio to assess whether order-level taxonomy influences the mid-point and width of crossover regions. Additionally, ANOVA was applied to determine if the origin of the parent sequences affects the mid-point and width of the crossover regions.

Distinct majority consensus sequences were identified from three datasets: (1) parent sequences that generated COI chimeric molecules in the full COI dataset, (2) parent sequences in the reduced COI dataset, and (3) parent sequences in the 18S rDNA dataset. A custom R script was then employed to analyze variation in GC content (Supplementary script 2) and DNA stability (ΔG°) (Supplementary script 3) along these consensus sequences using a sliding window approach. This analysis is important because high GC content and DNA stability hinders the ability of DNA polymerase to extend strands, creating potential hotspots for the formation of chimeras. This analysis employed a window size of 30 base pairs and a step size of 1 bp. ΔG° values in this analysis considered both base pairing (which relies on the hydrogen bonds between opposite nucleotides) and base stacking (which depends on the interactions between neighboring nucleotides on the same strand) [33]. Trends in ΔG° were modeled and visualized using a Generalized Additive Model (GAM) framework implemented with the R package *mgcv* ver. 1.9.1 [34]. GAM makes it possible to capture non-linear relationships between predictors and the response variable without assuming a specific relationship between the parameters.

A sliding window analysis with a window size of 5 base pairs and a step size of 1 base pair was used to determine if crossover regions in chimeric molecules coincided with regions of high sequence similarity between the parent molecules (Supplementary script 4). This approach was chosen because partially extended DNA strands require a few nucleotides of complementary sequence at their 3’ ends to efficiently bind to a different template [35]. To examine this matter, the percentage identity was determined for three datasets: (1) parent sequences that produced COI chimeric molecules in the full COI dataset, (2) parent sequences that produced chimeras in the reduced COI dataset, and (3) parent sequences that generated 18S rDNA chimeric molecules. A GAM function was employed to model the trends in percentage identity for both gene regions.

Custom R scripts used for the statistical analysis were developed with assistance from ChatGPT (OpenAI, San Francisco, CA, USA).

## Results

### Comparison of reduced COI and 18S rDNA datasets

This comparison examined the number of chimeras detected in the downsized COI and 18S rDNA datasets. For each gene region, 187,183 reads were analysed. The COI and 18S rDNA sequences for all 531 samples, along with their corresponding taxonomic assignments, are provided in the .xlsx file of Supplementary Table 2.

#### Sequence recovery

The comparison of sequence distribution across the 531 samples revealed a difference in recovery between COI and 18S rDNA. At least one COI sequence (either arthropod or nematode) was recovered from 521 of the 531 (98.1%) specimens with 402 (75.7%) producing two OTUs, 89 (16.8%) producing 3 or more OTUs, and 30 (5.6%) delivering just one OTU. By comparison, 18S rDNA was recovered from 518 of the 531 (97.6%) specimens with 273 (51.4%) producing two OTUs, 165 (31.1%) producing three or more OTUs, and 80 (15.1%) producing a single OTU (Supplementary Fig. 1).

**Fig. 1:**
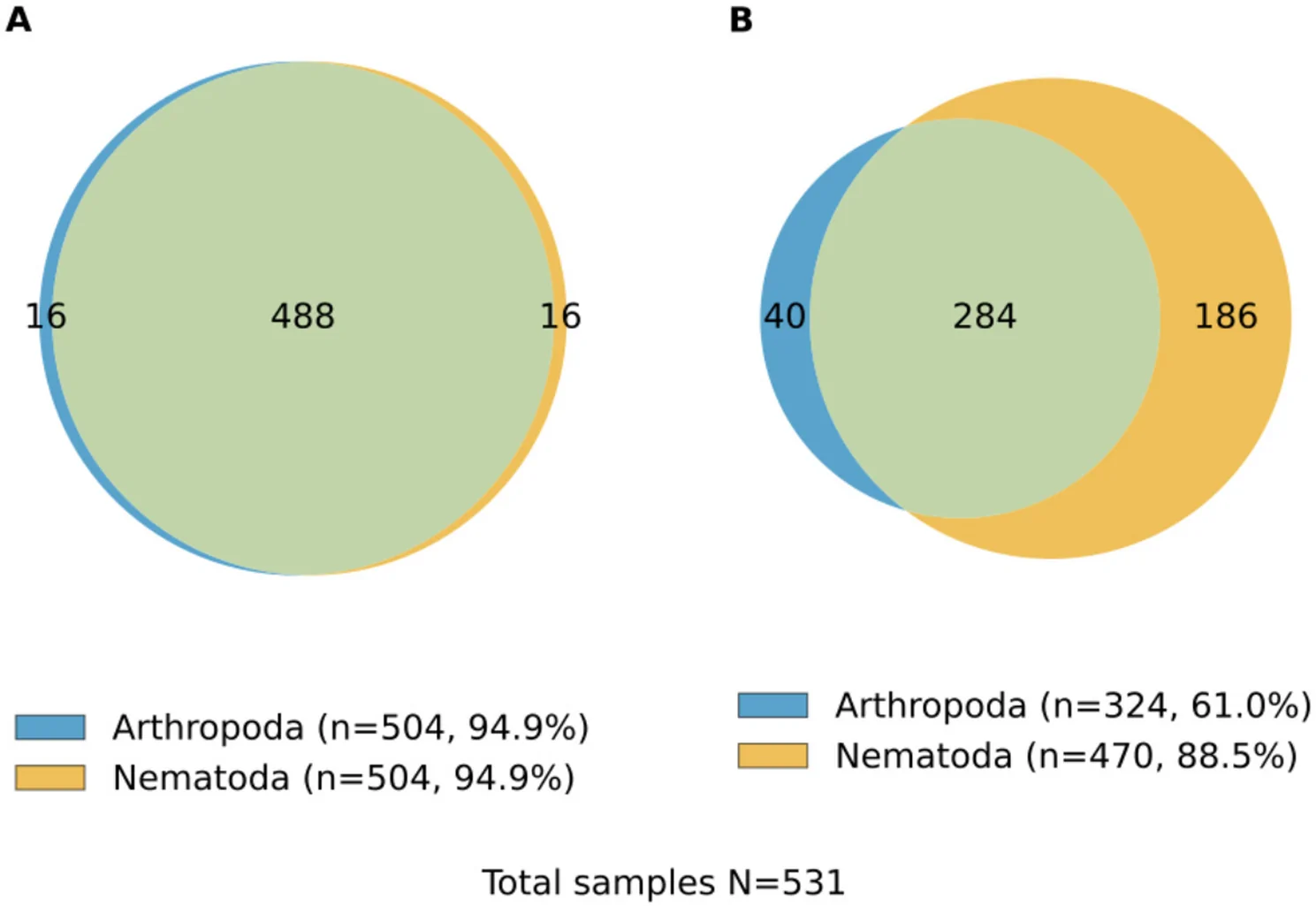
Venn diagram displaying sequence recovery for 531 arthropod specimens and associated nematodes assessed for the reduced COI (**A**) and 18S rDNA (**B**) datasets

Under ideal conditions, we would expect 531 arthropod and 531 nematode sequences from both COI and 18S rDNA markers. In practice, the same recovery success (94.9%) was achieved for COI with 504 arthropods and 504 nematode sequences. Four hundred and eighty-eight of the 531 specimens (91.9%) yielded both arthropod and nematode sequences, while 16 (3%) only yielded arthropod sequences and 16 (3.0%) only nematode sequences (Fig. 1A). 18S rDNA analysis recovered arthropod sequences from 324 specimens (53.5%) and nematode sequences from 470 (88.5%). Two hundred and eighty-four specimens (53.5%) yielded both arthropod and nematode sequences, 40 (7.5%) yielded arthropod sequences only, and 186 (35.0%) yielded nematode sequences only (Fig. 1B).

#### Detection of chimeras

The one-at-a-time parameter sensitivity analysis of *uchime_denovo* revealed that chimera detection was highly robust to parameter variation across all three datasets. For the reduced COI dataset, *uchime_denovo* at default settings detected 15 chimeric sequences. *Mindiffs* and *mindiv* had no effect on detection, *minh* had a negligible effect (lowering it to 0.1 added one sequence), while *abskew* was the most influential parameter: lowering it to 1.2 added three sequences (18 total) while raising it to 4.0 reduced detections by one (14 total). For 18S rDNA, *uchime_denovo* at default settings detected 173 chimeric sequences. *Minh*, *mindiffs*, and *mindiv* had no effect. *Abskew* was again the most influential parameter: lowering it to 1.2 added three sequences (176 total) while raising it to 4.0 reduced detections by 17 (156 total), representing the largest parameter effect observed across all datasets. For the full COI dataset, *uchime_denovo* at default settings detected 182 chimeric sequences. *Mindiffs* and *mindiv* had no effect, *minh* had a negligible effect (adding one chimera at its lowest setting), and *abskew* again produced the largest effect (183 at abskew = 1.2, 177 at abskew = 4.0) (Supplementary Table 3).

The *chimeras_denovo* algorithm produced identical results across all three segmentation configurations for all three datasets: 14 chimeric sequences for the reduced COI dataset, 124 for 18S rDNA, and 168 for the full COI dataset. This complete invariance to segmentation demonstrates that chimera detection was unaffected by fragment boundary placement and that no chimeras with breakpoints near sequence termini were missed (Supplementary Table 3).

Independent visual inspection of aligned sequences identified 11 chimeric sequences across 10 specimens of the reduced COI dataset, 122 across 93 specimens of the 18S rDNA dataset, and 138 across 113 specimens of the full COI dataset. Visual detection was restricted to chimeras exhibiting a single unambiguous crossover location (Supplementary Table 3).

The intersection of *uchime_denovo* detections at default parameters and independent visual inspection was used to define the final high-confidence chimera datasets for all subsequent analyses. For the reduced COI dataset, 9 sequences were confirmed by both methods across 8 specimens. For 18S rDNA, 119 sequences were confirmed across 91 specimens. For the full COI dataset, 137 sequences were confirmed across 112 specimens. All 9 chimeras identified in the reduced COI dataset were also recovered in the full COI consensus dataset, confirming the consistency of detection across sample sizes (Supplementary Table 3).

#### Characterization of crossover sites

**Fig. 2:**
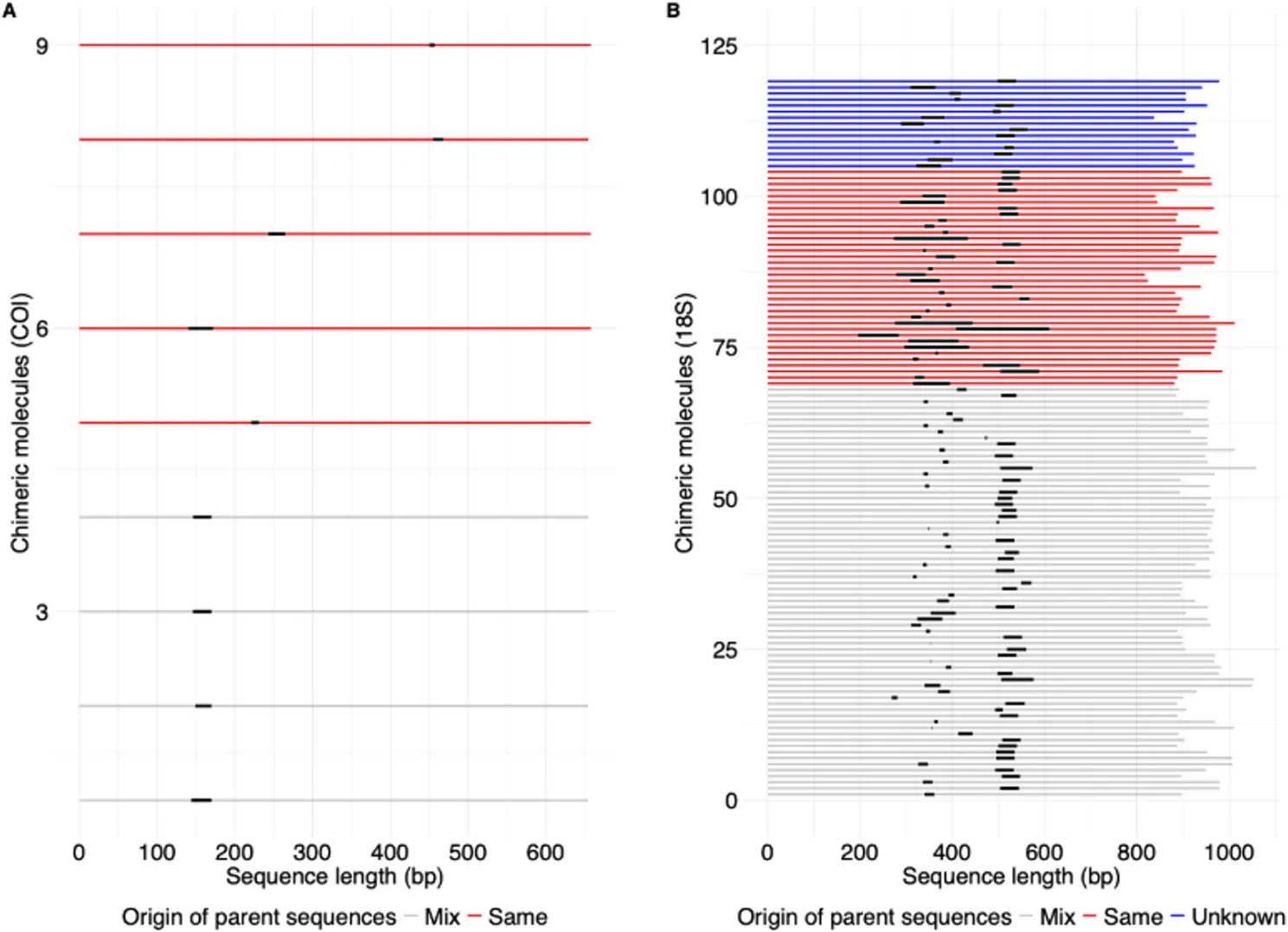
Location of the crossover ranges for the examined chimeric sequences for the reduced COI (**A**) and 18S rDNA (**B**) datasets. Each horizontal line represents a chimeric sequence, color-coded based on the classification of its parent sequences: Mix in grey (from different phyla), Same in red (from the same phylum), Unknown in blue (originating from unidentified phyla). Thick black lines indicate the crossover regions within each chimera

Analysis of the 9 COI chimeric molecules with a single crossover site showed crossovers in the central part of the sequence, between ~ 150 bp and ~ 460 bp (Fig. 2). Although the distribution of crossover midpoints suggested a bimodal pattern, the Hartigan’s Dip Test did not reject the null hypothesis of unimodality (D = 0.107, p = 0.431), indicating insufficient statistical evidence for multimodality (Supplementary Fig. 2). Similar to COI, analysis of crossover locations in the 119 chimeric 18S rDNA molecules characterized by a single crossover site showed that crossovers were centrally located, ~ 300–550 bp from the 5’ end. However, this central region contained two distinct crossover hotspots at ~ 300–400 base pairs, and at ~ 500–550 base pairs from the 5’ end (Fig. 2). Visual inspection of the Kernel density function confirmed these two distinct regions, revealing a bimodal distribution, further supported by Hartigan’s Dip test, which strongly rejected unimodality (D = 0.116, p-value < 0.001) (Supplementary Fig. 2). ANOVA revealed a significant effect of parent sequence category (i.e., same phylum, different phyla, unknown phylum) on the width of crossover regions in 18S rDNA chimeras (df = 2, F = 10.26, *p* < 0.001) (Supplementary Table 4). The taxonomic origins of the parent sequences show broadly similar patterns between COI and 18S rDNA (Fig. 2). For COI, within-phylum chimeras were slightly more common (5/9; 55.6%) than cross-phylum chimeras involving arthropod and nematode sequences (4/9; 44.4%). For 18S rDNA, this trend was reversed: arthropod-nematode chimeras predominated (68/119; 57.1%), followed by within-phylum pairs (36/119; 30.3%) and chimeras with one parent of unknown origin (15/119; 12.6%).

#### DNA stability and conserved regions

**Fig. 3:**
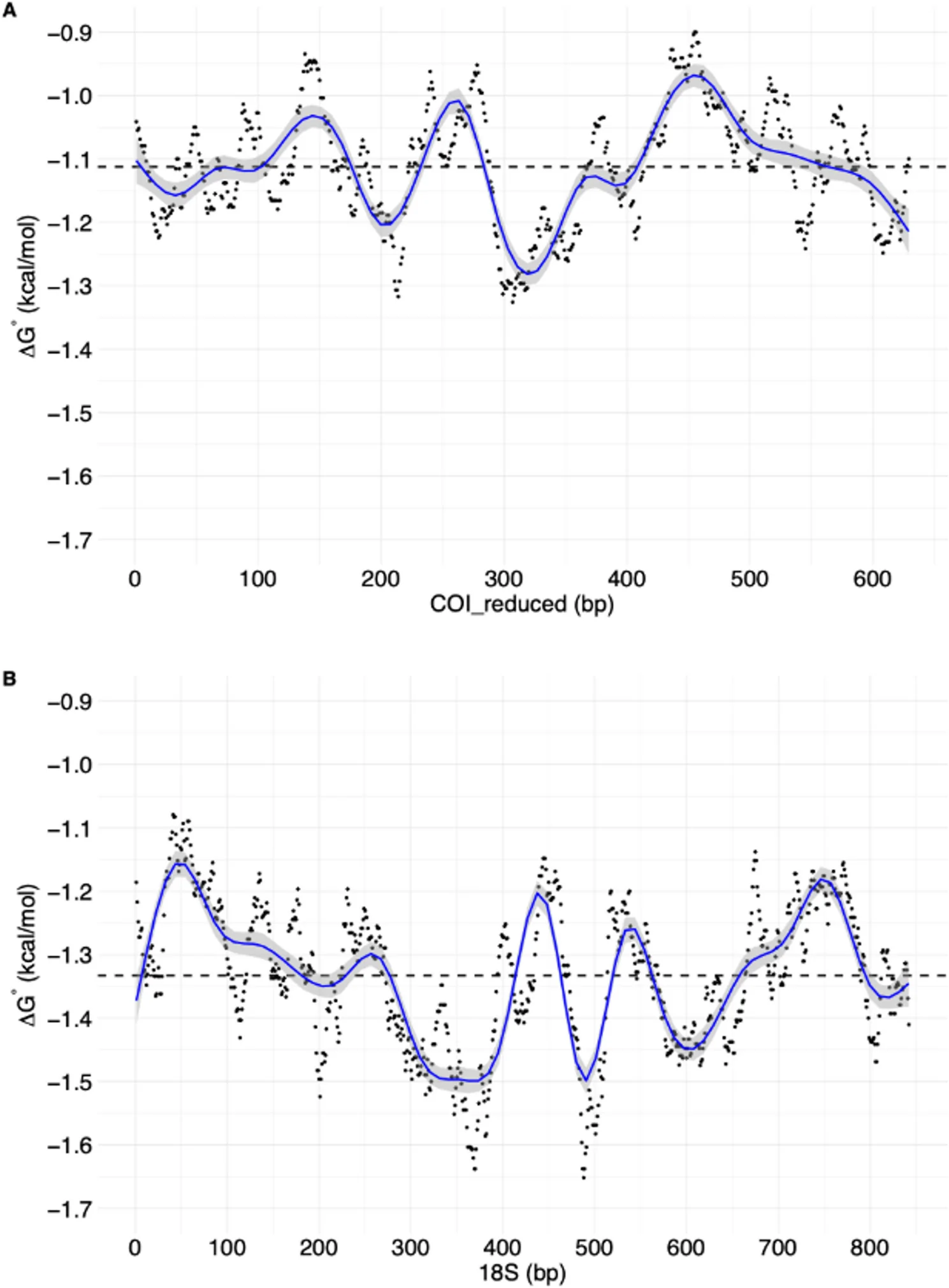
Sliding window analysis (window size = 30 bp; step size = 1 bp) of DNA stability (ΔG°) along majority consensus sequences derived from alignments of chimera parent sequences, for the reduced COI (**A**) and 18S rDNA (**B**) datasets. The average value is represented by the dotted black line; the trendline is modelled using a GAM function

The average GC content of the consensus sequence derived from the aligned parent sequences of the COI chimeras was 26%, considerably lower than the 44% for 18S rDNA (Supplementary Fig. 3). As expected, GC content displayed an inverse relationship with ΔG°, with regions of high GC content (and low ΔG°) indicating increased structural stability (Fig. 3). The consensus COI sequences had an average ΔG° of − 1.11 kcal/mol with the lowest values reaching − 1.34 kcal/mol (Fig. 3). By comparison, the consensus of the 18S rDNA sequences had an average ΔG° of − 1.33 kcal/mol, with two regions (~ 300–400 bp and ~ 500–550 bp from 5’ end) reaching − 1.65 kcal/mol (Fig. 3).

**Fig. 4:**
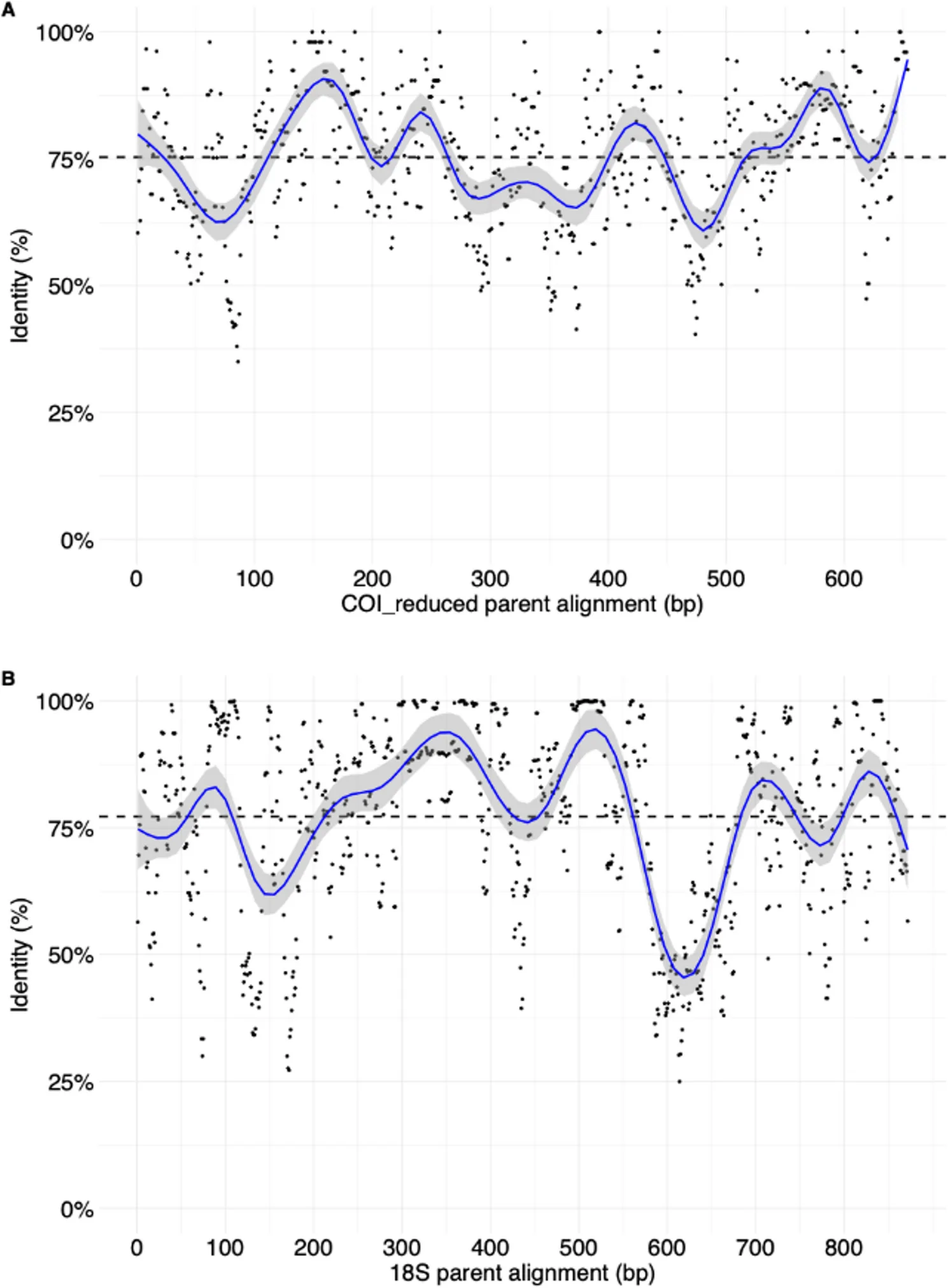
Sliding window analysis (window size = 5 base pairs and step size = 1 bp) examining percentage of identity from the alignments of chimera parent sequences for the reduced COI (**A**) and 18S rDNA (**B**) datasets. The dashed horizontal line represents the average percentage of identity; the trendline is modelled using a GAM function

The aligned parent sequences of the COI chimeras showed a fluctuating trend with a slight increase in identity (i.e., more conserved regions) at ~ 150–250 bp, at ~ 400–450 bp, around 600 bp, and at ~ 650 from the 5’ end. By contrast, the aligned parent 18S rDNA sequences revealed two regions where identity frequently reaches 100% (i.e., perfectly conserved regions) at ~ 300–400 bp and ~ 500–550 bp from the 5’ end (Fig. 4).

### Analysis of the full COI dataset

This analysis utilized the complete dataset of COI amplicons comprising a total of 1,486,303 reads.

#### Detection of chimeras and characterization of crossover sites

**Fig. 5:**
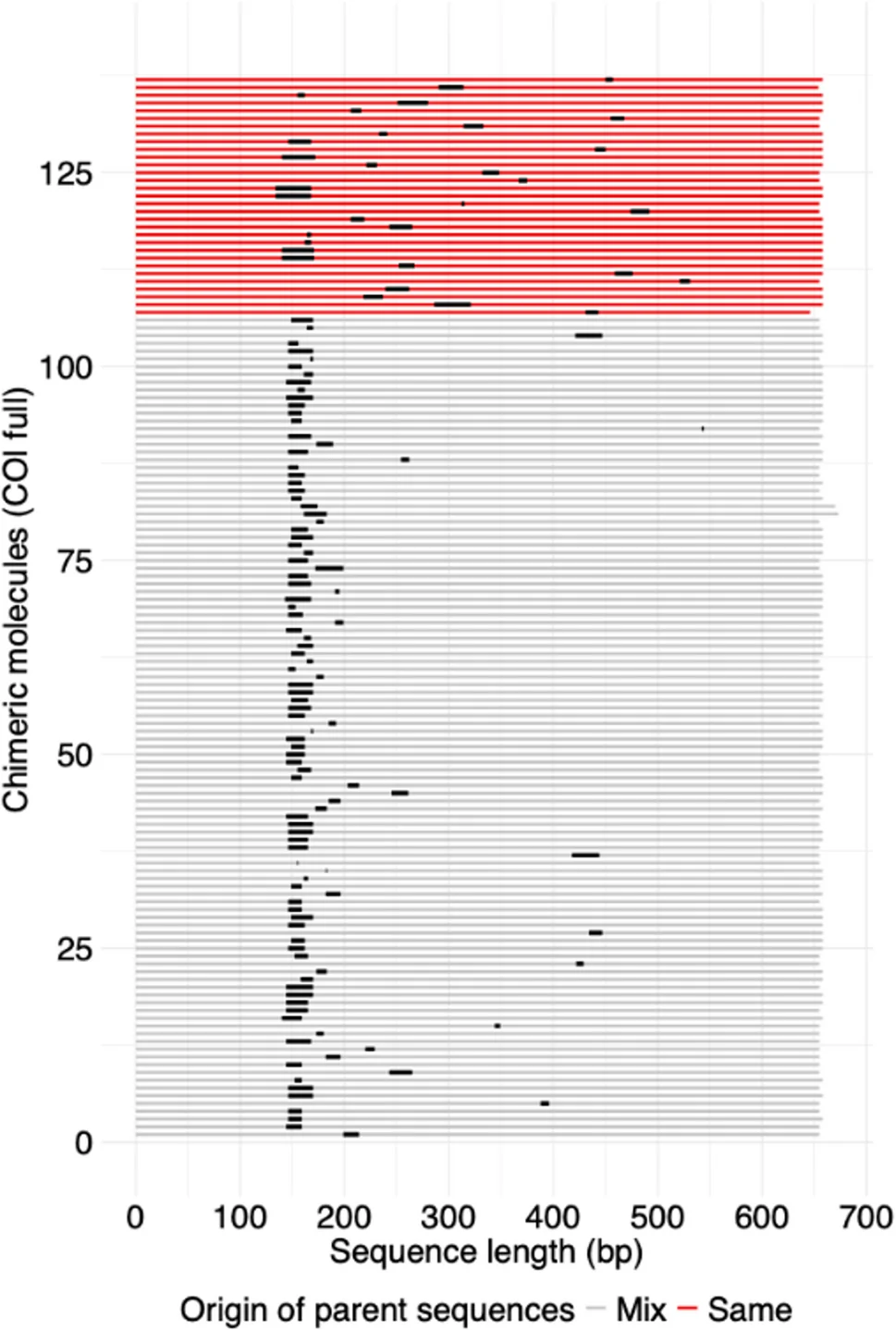
Location of the crossover ranges for the 137 chimeric sequences for the full COI dataset. Each horizontal line represents a chimeric sequence, color-coded based on the classification of its parent sequences: Mix (from different phyla), Same (from the same phylum); black thick lines represent crossover ranges

*uchime_denovo* at default settings identified 182 chimeric sequences of which 137 (75.3%) were confirmed visually. Analysis of these 137 COI chimeric molecules showed crossover sites spanning the sequence between ~ 150 bp and ~ 550 bp, but most (102 out of 137, 74%) were concentrated between ~ 150–200 bp. The remaining 35 crossovers (25.5%) were more sparsely distributed up to ~ 550. The Kernell density estimate and Hartigan’s dip test further supported this observation reveling a strongly unimodal distribution (D = 0.036; p-value = 0.211) with a right-skewed tail (Supplementary Fig. 4). The taxonomy of the parent sequences revealed that 106 of the 137 (77.4%) chimeric sequences were formed between an arthropod and a nematode, and 31 originated from parents belonging to the same phylum (22.6%) (Fig. 5).

ANOVA revealed that the mid-point of cross-over regions differed significantly by host organism (F = 3.46, *p* = 0.018) and by the source of parent sequences (F = 34.63, *p* < 0.001).

#### DNA stability and conserved regions

**Fig. 6:**
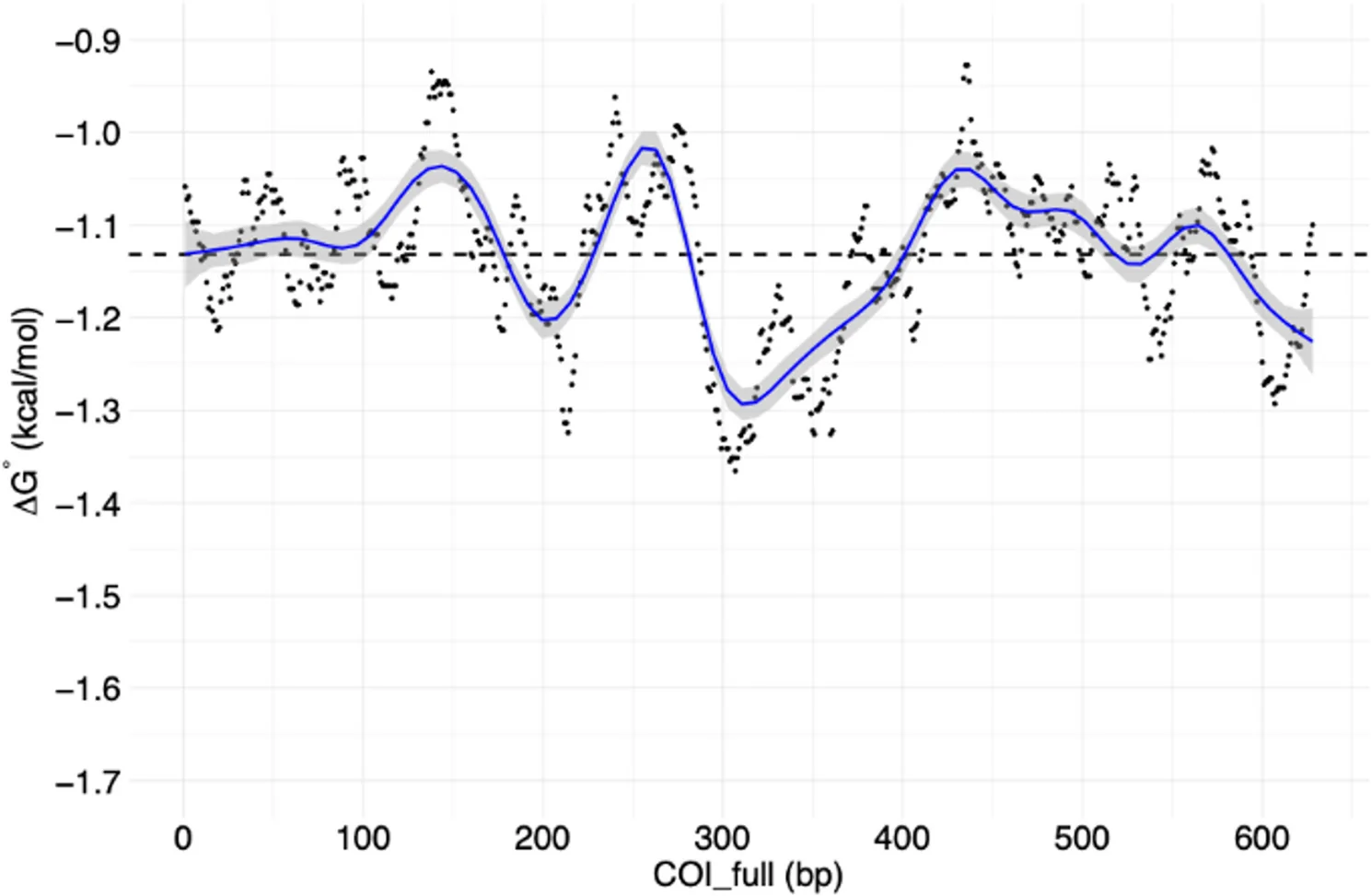
Sliding window analysis (window size = 30 base pairs and step size = 1 bp) of DNA stability (measured as ΔG°) in the majority consensus sequence of the full dataset of COI, derived from the alignments of parent sequences of the chimeras. The average value is represented by the dotted black line; the trendline is modelled using a GAM function

The consensus sequence for the aligned COI sequences had an average GC content of 26% (Supplementary Fig. 5) and an average ΔG° of − 1.13 kcal/mol (Fig. 6). Similar to the reduced COI and 18S rDNA datasets, GC content and ΔG° exhibited an inverse relationship. Regions with high DNA stability, characterized by increased GC content (Supplementary Fig. 5) and lower ΔG° values (Fig. 6), were observed near 200 bp, 300 bp, 350 bp, and 600 bp from the 5’ end.

**Fig. 7:**
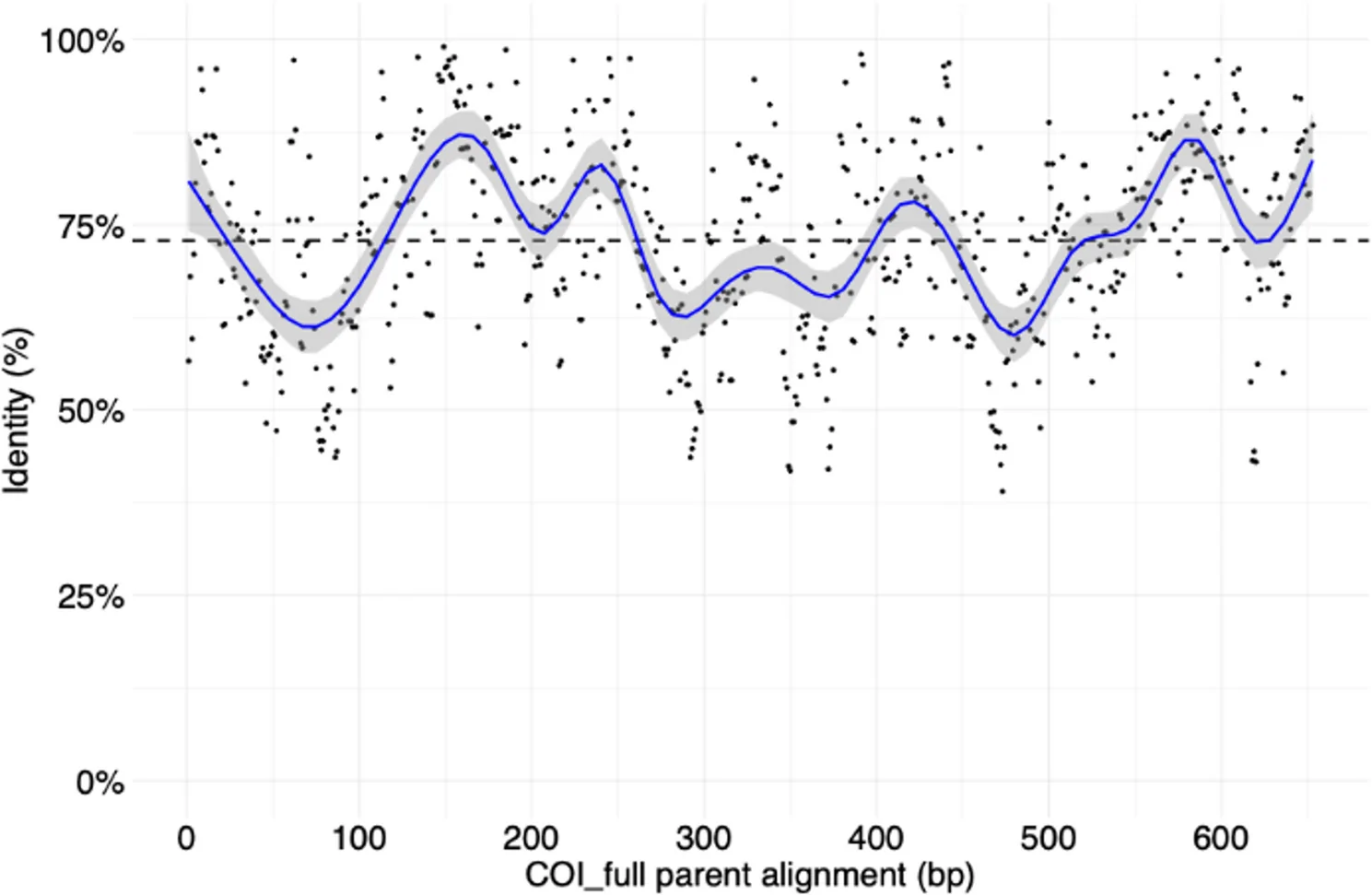
Sliding window analysis (window size = 5 base pairs and step size = 1 bp) examining percentage of identity from the alignments of chimera parent sequences for the full COI dataset. The dashed horizontal line represents the average percentage of identity; the trendline is modelled using a GAM function

The alignment of parent sequences for the COI chimeras displayed a variable pattern, with higher sequence identity (i.e., more conserved regions) between ~ 150–250 bp, just beyond ~ 400 bp, and between ~ 550–600 bp from the 5’ end (Fig. 7).

## Discussion

Sequencing of targeted gene regions is widely used to characterize species diversity. However, accurate estimates of diversity can be impeded by methodological artefacts. This study examines the frequency and nature of chimeric sequences generated during PCR amplification of two genes (COI, 18S rDNA) in DNA extracts from arthropods infected with nematodes.

The results revealed equal success in COI recovery from host arthropods and their nematode parasites (94.9%). This outcome was expected given that specimens were initially selected because their COI sequence arrays contained reads from both arthropod hosts and nematode parasites. In contrast, 18S rDNA recovery was considerably higher for nematodes (88.5%) than for arthropods (61%), likely reflecting the fact that the 18S rDNA primers were originally designed for nematodes [23]. The selection of appropriate genetic markers and primer pairs is a critical variable for eDNA metabarcoding and symbiome studies [36]. However, due to variation in target organisms, gene regions, PCR conditions, and sequencing platforms across studies, reaching general conclusions about marker taxonomic recovery is challenging. Nevertheless, several studies converge on the conclusion that COI and 18S rDNA provide complementary taxonomic coverage. In coastal macroinvertebrate communities, Leite et al. [37] found that ~ 69% of species were exclusively detected by either 18S rDNA or COI. In littoral hard-bottom communities, Wangensteen et al. [25] reported that COI recovered ~ 24% more arthropod MOTUs per sample than 18S rDNA, while 18S rDNA recovered ~ 10× more nematode MOTUs than COI. The same directional pattern was observed in deep-sea sediments by Atienza et al. [24], where Arthropoda was the most diverse phylum and Nematoda the second most diverse with COI, whereas the order reversed with 18S rDNA. Together, these studies indicate that the two markers preferentially amplify different segments of metazoan diversity, with Arthropoda biased toward COI recovery and Nematoda toward 18S rDNA recovery.

Chimeras can be prevalent [38], comprising up to 40% of PCR products in some studies [39, 40]. The software employed for their detection typically splits sequences into smaller fragments, which are then queried against a reference dataset of chimera-free sequences [18, 28] or analyzed through a *de novo* approach [28, 41, 42]. About a third of chimeras escape detection with these methods [39, 42], while approximately 2% are incorrectly identified as chimeras [41]. This underscores the need for more effective detection methods and a deeper understanding of both the frequency of chimeras and the factors contributing to their formation.

The present comparison revealed 13 times more chimeras for 18S rDNA than COI (119 versus 9 sequences confirmed by UCHIME default confirmed by visual assessment). Systematic variation of four key *uchime_denovo* parameters across nine configurations only produced minor changes in the number of chimeras detected. The minimum abundance ratio between parent sequences and candidate chimeras showed the greatest influence, accounting for approximately 27% and 12% of the variation in the detection of COI and 18S rDNA chimeras respectively. The minimum score threshold had a negligible effect on COI detection only, while the other parameters had no impact on either genetic marker. Given the much higher GC content of 18S rDNA (44%) than COI (26%), the elevated incidence of 18S rDNA chimeras is consistent with prior reports linking high GC content to chimera formation [38]. Despite this known association, the underlying reason(s) for it has never been examined. Our results suggest that two interconnected mechanisms influence chimera formation. First, the prerequisite for the genesis of chimeras is the formation of incompletely extended strands. While Taq DNA polymerase typically adds about 50 nucleotides per binding event [43], regions of high DNA stability increase the likelihood of polymerase dissociation [44]. This study supports this hypothesis by demonstrating the correspondence between regions of high DNA stability (i.e., low ΔG°) and crossover hotspots, with the 18S rDNA gene revealing hotspots for chimera formation between 300–400 bp and 500–550 bp from the 5’ end. Second, chimera formation requires that incompletely extended strands bind to a different template. Conserved regions are a critical enabler as the 3’ end of the incomplete strand must bind to template from another species and continue extension [45, 46]. This study revealed a correspondence between conserved regions and crossover hotspots, with 18S rDNA displaying two regions (300–400 bp, 500–550 bp) with close similarity between species of Arthropoda and Nematoda. Establishing a clear correlation between crossover hotspots, DNA stability, and conserved regions for the reduced COI dataset was challenging because so few chimeras were detected. Analysis of the full COI dataset did not reveal a correlation between DNA stability and crossover regions, but it did indicate a correlation between conserved regions and crossover hotspots, particularly near 150 bp from the 5′ end. This result suggests that sequence identity has a greater influence than DNA stability, particularly for genes like COI with low DNA stability throughout the sequence. This is further supported by the observation that many COI and 18S rDNA derived chimeras did not originate, as one might have expected, from the host arthropod and its parasitic nematode. Instead, they arose from either two arthropods or two nematodes. While some of these within-phylum combinations may reflect genuine biological interactions, the majority appear to have arisen as a result of contamination. Notably, no chimeras were detected near the termini of COI and 18S rDNA, a pattern that might have been an algorithmic artifact imposed by the standard four-segment division of *uchime_denovo*. However, careful visual inspection of sequence alignments identified no crossover locations near the termini of either gene. Importantly, the *chimeras_denovo* function, which employs a different scoring logic and allows user-defined segmentation, produced identical detection across 4-, 6-, and 8-segment configurations for both markers. Had segmentation boundaries constrained the detection of crossover locations, increasing the number of segments would have revealed new chimeras in previously inaccessible terminal regions, but no such change was observed. The absence of crossover locations near the termini likely reflects two distinct mechanisms. First, steady polymerase extension from its initial binding site on the template may reduce the likelihood of premature detachment and template switching. Second, early detachment of the polymerase during extension may still allow rebinding to the same template and a resumption of extension, generating a crossover-free window near the termini of the amplicons. If the latter mechanism plays a key role, an increased extension time would allow the polymerase to travel further before detachment, shifting this crossover-free window further into the amplicon. Both potential mechanisms need to be tested.

Examination of the full COI dataset revealed that the mid-point of cross-over regions differed significantly by host organism (F = 3.46, *p* = 0.018) and by the source of parent sequences (F = 34.63, *p* < 0.001). These two effects are likely not independent, as the host organism contributes one of the two parent sequences and thus partly determines their taxonomic source combination. The effect of both factors may therefore reflect taxon-specific sequence features of the parent sequences that favour or disfavour chimera formation at particular sites. The higher sequence conservation of 18S rDNA across taxa likely reduces taxon-specific sequence variation, resulting in cross-over events occurring at similar locations regardless of the taxonomic identity of the parent sequences. While COI chimeras showed no significant effect of parent sequence taxonomy on the width of the cross-over range, 18S rDNA chimeras revealed a highly significant effect (F = 10.26, *p* < 0.001). It should be noted that our analyses considered only whether the parent sequences belonged to the same or different phyla. For COI, the higher mutation rate may lead to sequence saturation, such that divergence between parent sequences is uniformly high regardless of their taxonomic source, consistently producing a narrow cross-over range. For 18S rDNA, the slower mutation rate results in lower sequence divergence within arthropod and within nematode lineages, making it difficult to precisely locate cross-over boundaries. By contrast, the high genetic divergence between these two phyla facilitates more precise identification of cross-over points, thereby widening the detectable crossover range.

It should be noted that the findings presented here are correlative in nature, derived from the characterization of chimeras arising in infected specimens, rather than from controlled experiments. As experiments using mock communities or spike-in controls with template sequences of known GC content, stability, and conservation could confirm the causal role for these factors in chimera formation, this would be valuable direction for future work.

## Conclusions

The formation of chimeras during PCR involves the concatenation of different template sequences, creating sequences absent from the original DNA extract. If unrecognized, these artefactual sequences can raise estimates of species diversity because both specialized parametric models [47] and general non-parametric approaches [48, 49] rely on observed diversity to estimate total diversity [50, 51]. In the present study, the oversight of chimeras would have inflated diversity by 0.8% for COI and by 11.2% for 18S rDNA. It needs emphasis that this study primarily focused on chimeras derived from organisms in different phyla. However, chimeras are both more frequent and much harder to detect when closely related species are involved [18, 41]. Detection gains further complexity in species with nuclear mitochondrial elements [52, 53], heteroplasmy [54, 55] or paralog genes [56] as chimeras can easily form between these intra-individual sequence variants. The present study advances our understanding of both the frequency of chimeras and the factors associated with their formation, such as DNA stability and the presence of highly conserved regions. These theoretical insights also have practical implications. Current chimera-detection software typically examines only the terminal regions of sequences or analyzes random fragments to identify potential chimeras. Incorporating information on crossover hotspots and examining their flanking regions will undoubtedly enhance detection accuracy, providing a more targeted and robust approach for identifying chimeric sequences.

## Supplementary Information

Supplementary Material 1: Supplementary figure 1. Histograms displaying the number of OTUs per sample detected for the reduced COI (A) and 18S (B) datasets.

Supplementary Material 2: Supplementary figure 2. Kernel density estimate of the mid-point values of the crossover ranges of chimeras for the reduced COI (A) and 18S (B) datasets. Hartigan's Dip Test statistic and p-value, testing for unimodality of the distribution, are shown above each plot.

Supplementary Material 3: Supplementary figure 3. Sliding window analysis (window size 30 base pairs and step size 1 base pair) of GC content in the majority consensus sequences derived from the alignment of parent sequences of the chimeras for the reduced COI (A) and 18S (B) datasets. Average value is represented by the dotted black line; the trendline is modelled using a GAM function.

Supplementary Material 4: Supplementary figure 4. Kernel density estimate of the mid-point values of the crossover ranges of chimeras for the full COI dataset. Hartigan's Dip Test statistic and p-value, testing for unimodality of the distribution, are shown above the plot.

Supplementary Material 5: Supplementary figure 5. Sliding window analysis (window size 30 base pairs and step size 1 base pair) of GC content in the majority consensus sequence of the full COI dataset, derived from the alignments of parent sequences of the chimeras. Average value is represented by the dotted black line; the trendline is modelled using a GAM function.

Supplementary Material 6: Supplementary table 1. List of samples with corresponding PAD sequences, UMIs (Unique Molecular Identifiers), and primer sequences, for both COI and 18S.

Supplementary Material 7: Supplementary table 2. List of samples with corresponding COI and 18S sequences, including taxonomic classification and sequence read counts.

Supplementary Material 8: Supplementary Table 3. Chimera detection results across the reduced COI, 18S, and full COI datasets. Each row represents a unique chimeric sequence, identified by Sample ID, operational taxonomic unit (OUT) designation, read count, and taxonomic assignment (Phylum, Class, Order). Detection outcome (1 = chimera detected, 0 = not detected) is reported for uchime_denovo across a one-at-a-time parameter sensitivity analysis, and for independent visual inspection. The column High-confidence_uchime_visual indicates sequences confirmed as chimeric by both uchime_denovo at default parameters and visual assessment.

Supplementary Material 9: Supplementary table 4. Analysis of variance (ANOVA) examining differences in metrics such as mid-point of crossover regions and width of crossover ranges among different categories such as host organism and source of the parent sequences for the COI reduced, 18S, and COI full datasets.

Supplementary Material 10: Supplementary script 1. R script used to display the crossover range of examined chimeric sequences and to color-code them based on the origin of their parental sequences.

Supplementary Material 11: Supplementary script 2. R script performing a sliding window analysis (30 bp window size, 1 bp step size) of GC content to estimate the percentage of sequence identity within a single alignment.

Supplementary Material 12: Supplementary script 3. R script that performs a sliding window analysis (30 bp window size, 1 bp step size) to estimate DNA stability, measured as ΔG°, in the majority consensus sequences of the parental molecules of chimeras.

Supplementary Material 13: Supplementary script 4. R script performing a sliding window analysis (5 bp window size, 1 bp step size) to estimate percentage of identity in the majority consensus sequences of the parental molecules of chimeras.

## Data Availability

The array of arthropod and nematode COI and 18S sequences derived from the 531 records are available on BOLD (https://boldsystems.org/) in the public dataset “DS-CHIMERA” (dx.doi.org/10.5883/DS-CHIMERA), and are provided in Supplementary Table 2.
