# Supplementary material for: Incidence and attributes of chimeric COI and 18S rDNA sequences derived from nematode-infected arthropods
Source: BMC Genomics. 2026 Aug 5;27:906. doi: 10.1186/s12864-026-13235-3 (PMC13726089; doi:10.1186/s12864-026-13235-3)
Supplement: Supplementary file 1 [file 12864_2026_13235_MOESM1_ESM.pdf]

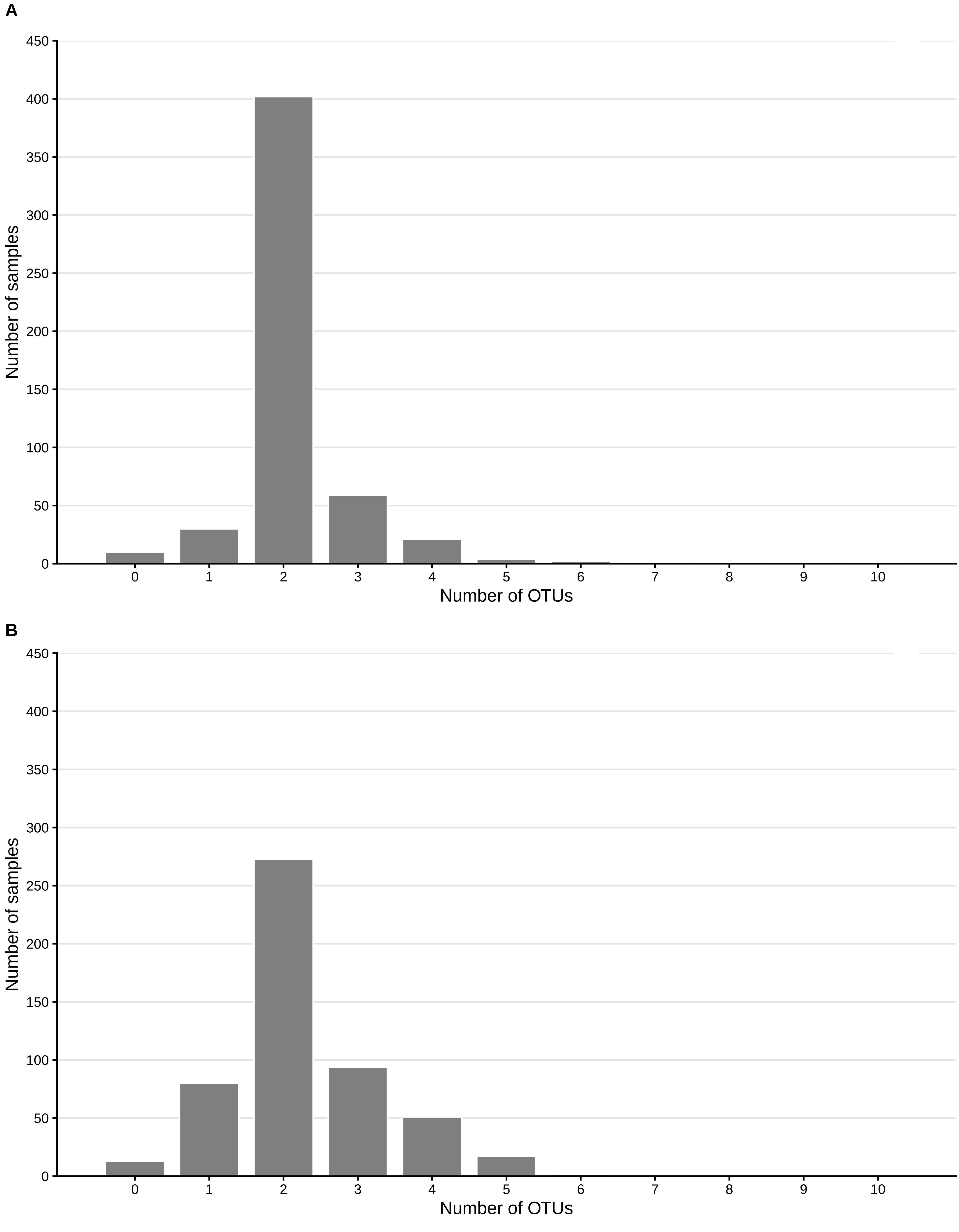
A
Number of samples
450
400
350
300
250
200
150
100
50
0
0
1
2
3
4
5
6
7
8
9
10
Number of OTUs
B
Number of samples
450
400
350
300
250
200
150
100
50
0
0
1
2
3
4
5
6
7
8
9
10
Number of OTUs
