# Supplementary material for: Incidence and attributes of chimeric COI and 18S rDNA sequences derived from nematode-infected arthropods
Source: BMC Genomics. 2026 Aug 5;27:906. doi: 10.1186/s12864-026-13235-3 (PMC13726089; doi:10.1186/s12864-026-13235-3)
Supplement: Supplementary file 2 [file 12864_2026_13235_MOESM2_ESM.pdf]

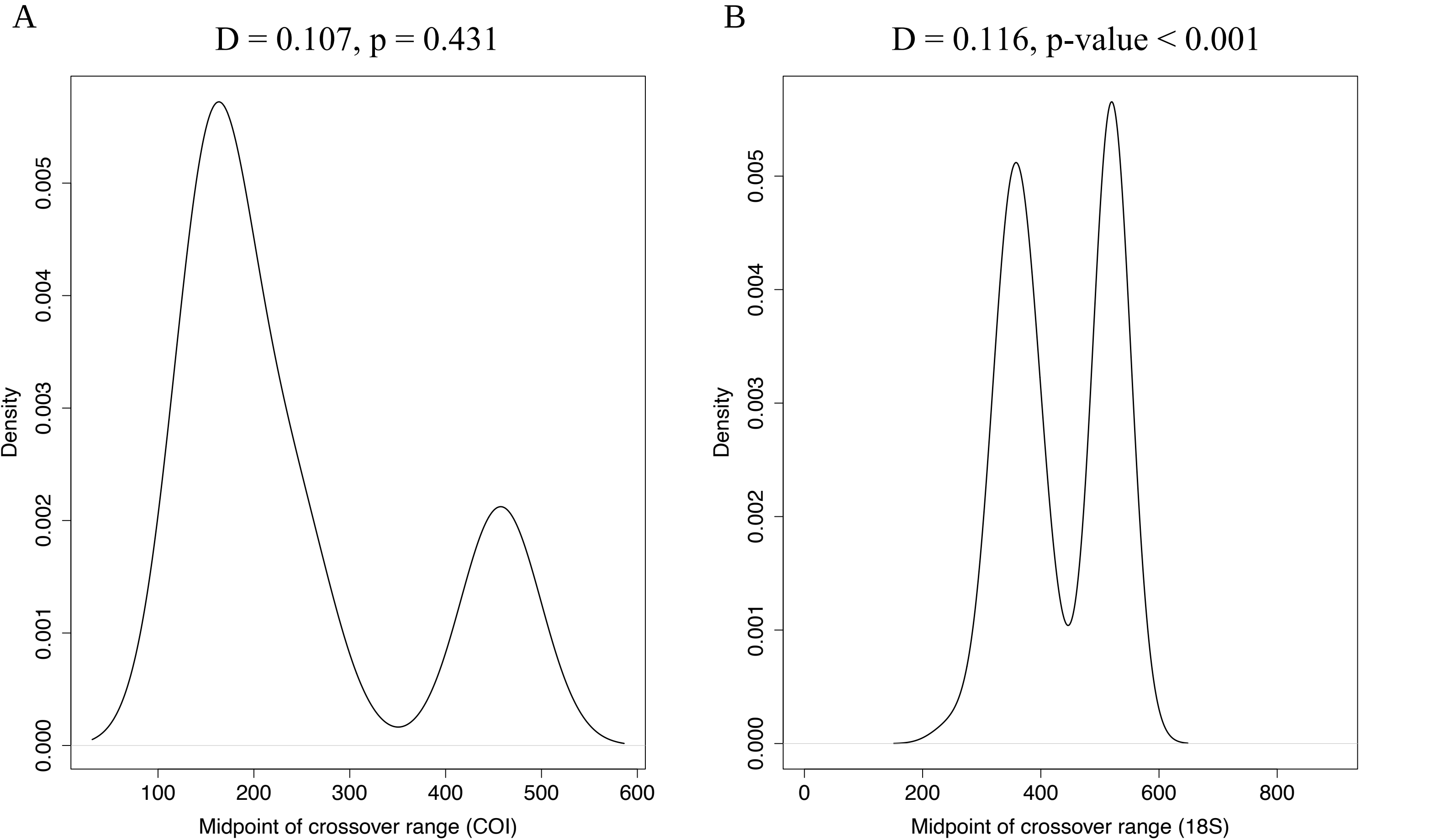

A
D = 0.107, p = 0.431
Density
0.000
0.001
0.002
0.003
0.004
0.005
100
200
300
400
500
600
Midpoint of crossover range (COI)
B
D = 0.116, p-value < 0.001
Density
0.000
0.001
0.002
0.003
0.004
0.005
0
200
400
600
800
Midpoint of crossover range (18S)
