# Supplementary material for: Incidence and attributes of chimeric COI and 18S rDNA sequences derived from nematode-infected arthropods
Source: BMC Genomics. 2026 Aug 5;27:906. doi: 10.1186/s12864-026-13235-3 (PMC13726089; doi:10.1186/s12864-026-13235-3)
Supplement: Supplementary file 3 [file 12864_2026_13235_MOESM3_ESM.pdf]

A

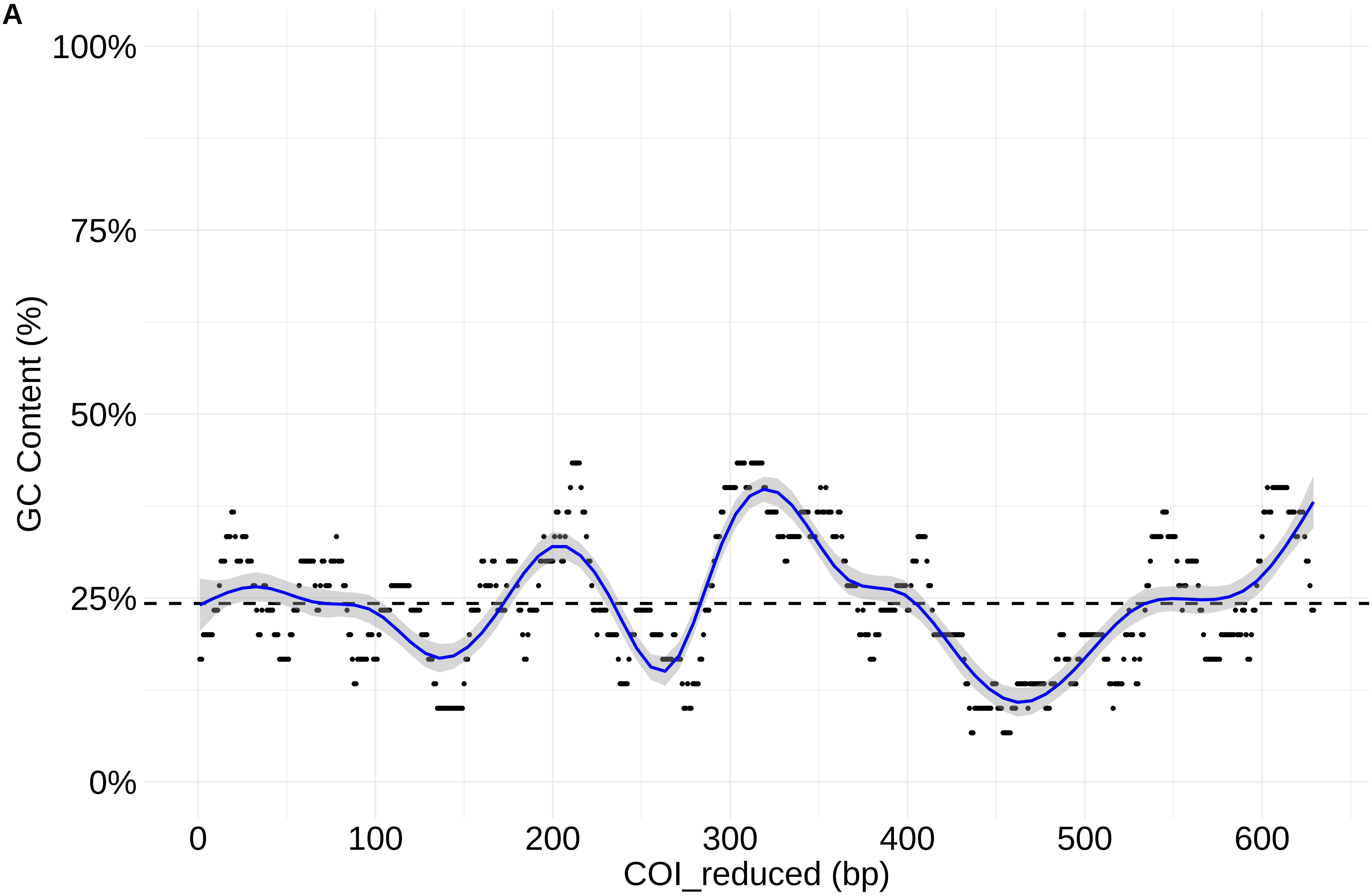

100%
75%
50%
25%
0%
GC Content (%)
0
100
200
300
400
500
600
COI_reduced (bp)

B

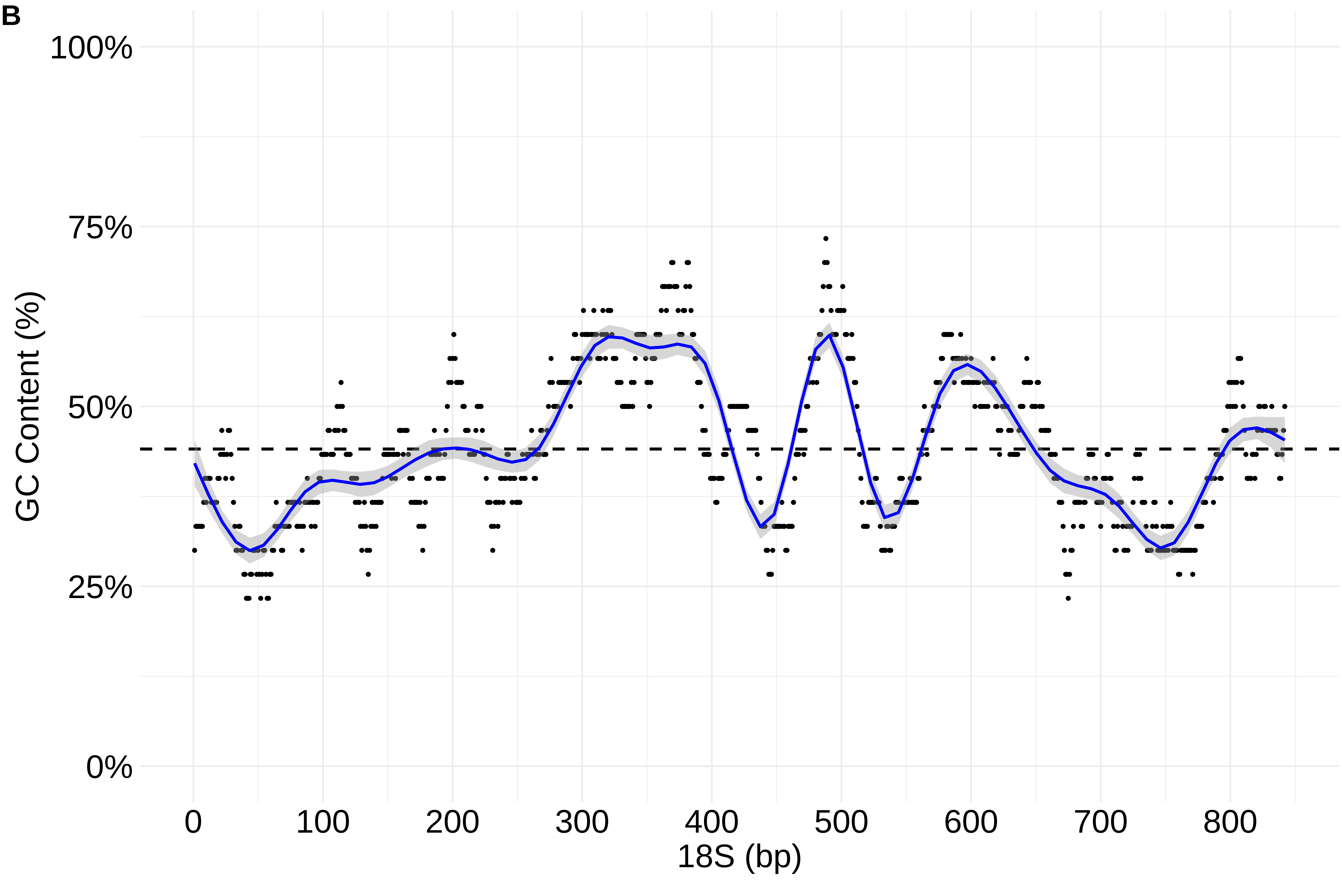

100%
75%
50%
25%
0%
GC Content (%)
0
100
200
300
400
500
600
700
800
18S (bp)
