# Supplementary material for: Incidence and attributes of chimeric COI and 18S rDNA sequences derived from nematode-infected arthropods
Source: BMC Genomics. 2026 Aug 5;27:906. doi: 10.1186/s12864-026-13235-3 (PMC13726089; doi:10.1186/s12864-026-13235-3)
Supplement: Supplementary file 4 [file 12864_2026_13235_MOESM4_ESM.pdf]

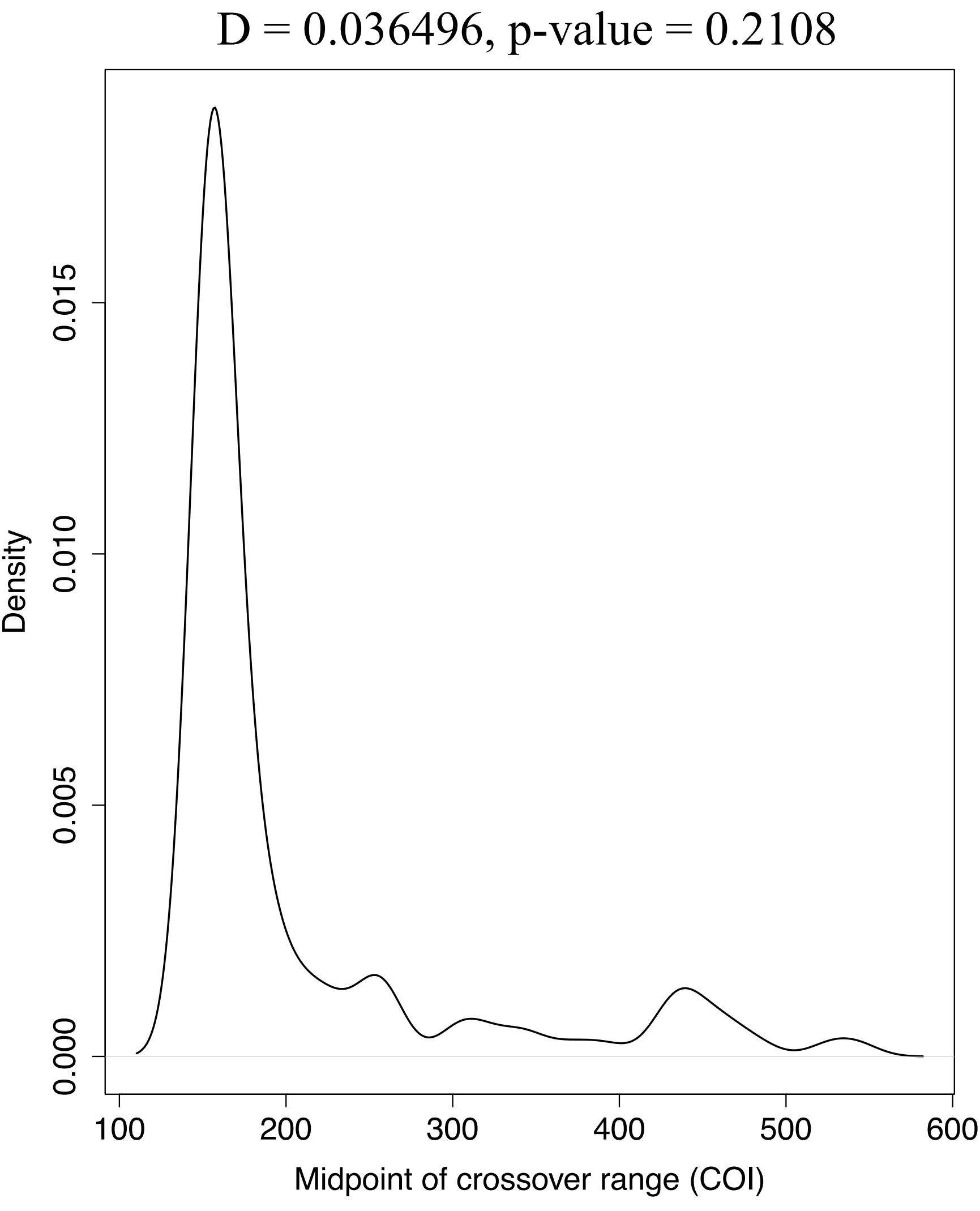
D = 0.036496, p-value = 0.2108
Density
0.000
0.005
0.010
0.015
100
200
300
400
500
600
Midpoint of crossover range (COI)
