# Supplementary material for: Incidence and attributes of chimeric COI and 18S rDNA sequences derived from nematode-infected arthropods
Source: BMC Genomics. 2026 Aug 5;27:906. doi: 10.1186/s12864-026-13235-3 (PMC13726089; doi:10.1186/s12864-026-13235-3)
Supplement: Supplementary file 6 [file 12864_2026_13235_MOESM10_ESM.docx]

#Graphical representation of crossover ranges and parental origin of chimeric sequences

\# Required libraries

library(readxl)

library(ggplot2)

\# Input file description:

\# The Excel file should contain the following columns:

\# - 'recomb_range': a string of the form "start-end" (e.g., "145-202")

\# - 'seq_length': total length of the chimeric molecule in base pairs

\# - 'system': one of "Unknown", "Same", or "Mix", describing the origin of the parent sequences

\# Step 1: Read Excel file

file_path <- file.choose() # Prompt user to select the input Excel file

dataset <- read_excel(file_path)

\# Step 2: Extract recombination breakpoints

start_end <- sapply(dataset$recomb_range, function(x) {

as.numeric(unlist(strsplit(x, "-")))

})

dataset$start <- start_end[1, ]

dataset$end <- start_end[2, ]

\# Step 3: Set plotting order of systems

dataset$system <- factor(dataset$system, levels = c("Mix", "Same", "Unknown"))

dataset <- dataset[order(dataset$system), ]

\# Step 4: Define plot colors

colors <- c("Mix" = "grey77", "Same" = "red2", "Unknown" = "blue")

\# Step 5: Create the plot

\# Note: adjust scale_y_continuous() breaks/limits to suit the number of

\# chimeric molecules in your dataset.

plot <- ggplot(dataset, aes(y = seq_along(seq_length), x = seq_length, color = system)) +

geom_segment(aes(yend = seq_along(seq_length), x = 0, xend = seq_length), size = 1) + # Full sequence line

geom_segment(aes(x = start, xend = end, yend = seq_along(seq_length)), color = "black", size = 1.5) + # Recombination region

scale_color_manual(values = colors, drop = TRUE) +

scale_x_continuous(breaks = seq(0, 1050, by = 100)) +

scale_y_continuous(breaks = seq(0, nrow(dataset), by = 3)) +

labs(

y = "Chimeric molecules",

x = "Sequence length (bp)",

color = "Origin of parent sequences"

) +

theme_minimal() +

theme(

legend.position = "bottom",

axis.title = element_text(size = 23, color = "black"),

axis.text = element_text(size = 23, color = "black"),

legend.title = element_text(size = 23, color = "black"),

legend.text = element_text(size = 23, color = "black"),

plot.margin = margin(20, 20, 20, 20)

)

\# Step 6: Display and save the plot

print(plot)

ggsave("crossover_plot.pdf", plot, width = 9, height = 13.5)
