# Supplementary material for: Incidence and attributes of chimeric COI and 18S rDNA sequences derived from nematode-infected arthropods
Source: BMC Genomics. 2026 Aug 5;27:906. doi: 10.1186/s12864-026-13235-3 (PMC13726089; doi:10.1186/s12864-026-13235-3)
Supplement: Supplementary file 9 [file 12864_2026_13235_MOESM13_ESM.docx]

\# Sliding window analysis of sequence identity

\# This script performs a sliding window analysis on sequence identity values.

\# The input file must be a CSV with two columns: 'Position' (numeric) indicating

\# the alignment position, and 'Identity' (numeric) representing pairwise identity

\# values at that position (ranging from 0 to 1).

\# Load required libraries

library(ggplot2)

library(mgcv)

library(scales)

\# ----- Define genetic marker (used only for axis labeling) -----

marker_name <- "18S" # Change as needed (e.g., "COI", "18S")

\# Prompt user to select the identity CSV file

cat("Please select the identity CSV file...\n")

file_path <- file.choose()

\# Read the selected file

identity_data <- read.csv(file_path)

\# Define sliding window parameters

window_size <- 5

step_size <- 1

\# Initialize result container

num_rows <- nrow(identity_data)

results <- data.frame(position = numeric(0), average_identity = numeric(0))

\# Perform sliding window calculation

for (i in 1:(num_rows - window_size + 1)) {

start <- i

end <- i + window_size - 1

window_identity <- mean(identity_data$Identity[start:end])

results <- rbind(results, data.frame(position = identity_data$Position[start], average_identity = window_identity))

}

avg_identity <- mean(results$average_identity, na.rm = TRUE)

\# ----- Define x-axis label -----

x_label <- paste0(marker_name, " parent alignment (bp)")

\# Plot results

plot <- ggplot(results, aes(x = position, y = average_identity)) +

geom_point(size = 1, color = "black") +

geom_hline(yintercept = avg_identity, linetype = "dashed", color = "black", size = 1) +

geom_smooth(method = "gam", formula = y ~ s(x, bs = "cs", k = 20), color = "blue") +

labs(

x = x_label,

y = "Identity (%)"

) +

scale_x_continuous(breaks = seq(0, max(identity_data$Position), by = 100)) +

scale_y_continuous(labels = percent_format(accuracy = 1), limits = c(0, 1)) +

theme_minimal() +

theme(

axis.title = element_text(size = 23, color = "black"),

axis.text = element_text(size = 23, color = "black"),

plot.margin = margin(20, 20, 20, 20)

)

print(plot)

ggsave(paste0(marker_name, "_identity.pdf"), plot, width = 13.5, height = 9)

cat("Average identity:", round(avg_identity * 100, 2), "%\n")
